# Quantification of Digital Body Maps for Pain: Development and Application of an Algorithm for Generating Pain Frequency Maps

**DOI:** 10.2196/36687

**Published:** 2022-06-24

**Authors:** Abhishek Dixit, Michael Lee

**Affiliations:** 1 Division of Anaesthesia Department of Medicine University of Cambridge Cambridge United Kingdom

**Keywords:** Scalable Vector Graphics, SVG, pain drawing, pain location, Body Pain Map, overlap computation, heat map, pain frequency map, algorithm

## Abstract

**Background:**

Pain is an unpleasant sensation that signals potential or actual bodily injury. The locations of bodily pain can be communicated and recorded by freehand drawing on 2D or 3D (manikin) surface maps. Freehand pain drawings are often part of validated pain questionnaires (eg, the Brief Pain Inventory) and use 2D templates with undemarcated body outlines. The simultaneous analysis of drawings allows the generation of pain frequency maps that are clinically useful for identifying areas of common pain in a disease. The grid-based approach (dividing a template into cells) allows easy generation of pain frequency maps, but the grid’s granularity influences data capture accuracy and end-user usability. The grid-free templates circumvent the problem related to grid creation and selection and provide an unbiased basis for drawings that most resemble paper drawings. However, the precise capture of drawn areas poses considerable challenges in producing pain frequency maps. While web-based applications and mobile-based apps for freehand digital drawings are widely available, tools for generating pain frequency maps from grid-free drawings are lacking.

**Objective:**

We sought to provide an algorithm that can process any number of freehand drawings on any grid-free 2D body template to generate a pain frequency map. We envisage the use of the algorithm in clinical or research settings to facilitate fine-grain comparisons of human pain anatomy between disease diagnosis or disorders or as an outcome metric to guide monitoring or discovery of treatments.

**Methods:**

We designed a web-based tool to capture freehand pain drawings using a grid-free 2D body template. Each drawing consisted of overlapping rectangles (Scalable Vector Graphics *<rect>* elements) created by scribbling in the same area of the body template. An algorithm was developed and implemented in Python to compute the overlap of rectangles and generate a pain frequency map. The utility of the algorithm was demonstrated on drawings obtained from 2 clinical data sets, one of which was a clinical drug trial (ISRCTN68734605). We also used simulated data sets of overlapping rectangles to evaluate the performance of the algorithm.

**Results:**

The algorithm produced nonoverlapping rectangles representing unique locations on the body template. Each rectangle carries an overlap frequency that denotes the number of participants with pain at that location. When transformed into an HTML file, the output is feasibly rendered as a pain frequency map on web browsers. The layout (vertical-horizontal) of the output rectangles can be specified based on the dimensions of the body regions. The output can also be exported to a CSV file for further analysis.

**Conclusions:**

Although further validation in much larger clinical data sets is required, the algorithm in its current form allows for the generation of pain frequency maps from any number of freehand drawings on any 2D body template.

## Introduction

### Background

Pain is an unpleasant sensation signaling potential or actual injury to the body site [1]. The location of pain can be communicated or recorded by drawing onto a body template (eg, Brief Pain Inventory [2]). Pain drawings have been used clinically and in research for decades [3]. Jang et al [4] reported that patients were more confident about communicating the locations of pain to the clinician in the form of a drawing as opposed to a written description, and the clinicians also favored drawings over written descriptions. Digital technology is now commonplace and circumvents problems associated with the processing and storage of pain drawings; hence, pain drawings are now widely acquired as digital images when possible [5]. Numerous web-based applications and mobile apps offer digital pain manikins, both commercial and academic [6]. Most digital pain manikins use 2D templates, which are whole-body coronal or sagittal views of the human body. There is often a choice of gender and body type for 2D or 3D surface templates [7-9]. A typical digital pain manikin consists of a body template and self-explanatory instructions for the patient regarding how to indicate the locations where they experience the most discomfort.

The pain frequency map is generated by the simultaneous analysis of all digital pain drawings to compute the locations of pain that participants have in common. To aid visualization, the maps also use color codes to highlight locations on the body in accordance with their frequency of occurrence in the sample being studied. Such maps are clinically useful in identifying where sensations commonly relate to disease anatomy [10,11] and the factors that influence the subjective localization of pathology. These maps are perhaps most important in chronic primary pain syndromes [12], which are defined by the locations of pain in the absence of disease (eg, chronic back pain).

The ease with which pain frequency maps are generated critically depends on the nature of the body template used. For body templates where the anatomical locations are already predefined and demarcated [7,13], the pain drawings are swiftly completed, and the data captured are binary, where *1* indicates a selection, and *0* indicates no selection. As a result, the generation of pain frequency maps is relatively straightforward, because only the number of participants who selected each location needs to be deduced [14]. However, such frequency maps may not entirely capture the underlined spatial patterns of pain because of loss of spatial resolution caused due to the pixel (ie, x-y coordinate, representing the smallest possible division for the body template) being preassigned to larger (hence fewer) anatomical regions (eg, the Collaborative Health Outcomes Information Registry [CHOIR] body template has 74 divisions to click [7]). In addition, assumptions must be made about where the anatomical locations of pain are clinically relevant or important.

*Freehand* pain drawings, using body templates with undemarcated or blank body outlines, are unbiased. Some body templates supporting freehand drawings use a grid with a predefined number of cells (eg, GeoPain app with a 3D body template uses a grid of 2026 cells [9] and Manchester Digital Pain Manikin uses a grid of 12,800 cells for its 2D body template [15]). The *grid*-*based* templates also allow the easy generation of pain frequency maps because each location (a cell) is binary in nature and is either selected or not selected. However, as body templates come in a variety of shapes and sizes [5], the aspect ratio (ratio of width to height) of the body regions also changes, and it is therefore not possible to standardize a grid (no *one-size-fits-all*). The optimal grid granularity (resolution) of each body template must be assessed. The granularity of the grid determines the end-user experience and accuracy of the captured data [15].

The *grid*-*free* body template, as the name suggests, overcomes the problem of grid creation and selection and provides an assumption-free basis for pain drawings that most resemble paper drawings. The number of clickable locations is of the order of thousands (depending on the pencil size). In addition, the participant may choose to draw repeatedly in the same location, similar to what happens when pen and paper are used. The precise capture of drawn areas, along with no predefined locations (cells), poses considerable challenges in generating pain frequency maps. The drawings on grid-free templates require intricate pixel-level analysis to generate a pain frequency map, and the tools for generating such maps are lacking or not freely available [16-18].

### Objectives

In this paper, we describe a novel and unbiased algorithm developed specifically to generate a spatial pain frequency map from freehand pain drawings on a generic 2D whole-body template. We also assessed the performance of the algorithm’s Python script and demonstrated its utility by generating pain frequency maps from pain drawings obtained from 2 clinical data sets.

## Methods

### Creating the Digital Body Template (Manikin)

An image outline (or template) of the human body is embedded in an HTML page to create a digital body template (manikin) to capture drawings of pain locations.

The responsiveness of the body template (ie, the ability to highlight or zoom in or out of a region) requires vectors, which are created using lines, points, and shapes to represent the different regions (demarcations) of the body. Using Scalable Vector Graphics (SVG), an XML-based language, it is possible to display vectors and create a responsive body template.

The locations of pain can be recorded on any body template by inserting basic SVG elements such as circles and rectangles. These shapes are inserted by specifying their position and size as the core attributes. For example, the circle requires coordinates (*cx*, *cy*) of the center and the radius (*r*), and the rectangle requires coordinates (*x*, *y*) of the top left corner along with *width* and *height*. In addition to the core attributes, the SVG elements can also contain style-related attributes (eg, fill, visibility, and opacity) and any number of customized attributes with prefix *data-* (eg, data-region and data-date-inserted).

The circle and rectangle elements can be created in SVG:

<circle cx=“5” cy=“5” r=“10” fill=“green” visibility=“hidden” />

<rect x=“5” y=“5” width=“20” height=“10” fill=“red” opacity=“0.8” data-region=“front” />

We used the SVG *<rect>* element to record the location of pain because the intersection of the 2 rectangles is always a rectangle (consistent geometry) that is required for the algorithm to function.

**Figure 1:**
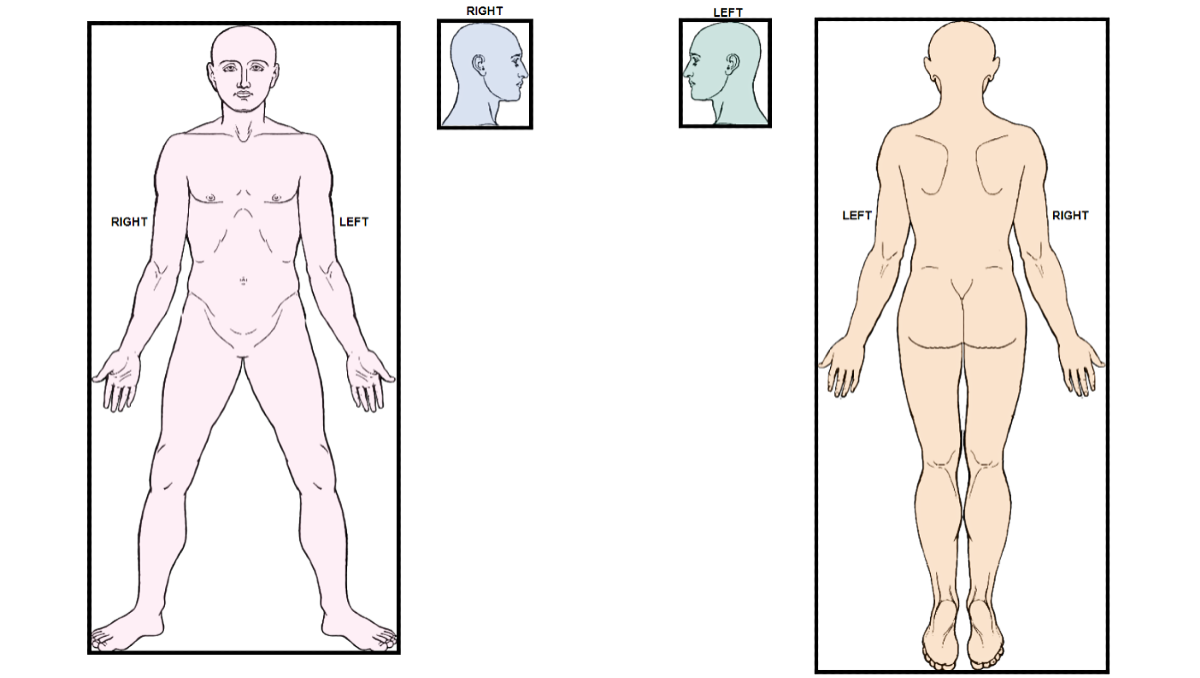
The body template (manikin) shows 4 regions. The tightest fitted rectangle (bounding box) enclosing each region is shown in black.

We downloaded a sexless human body outline image (TIFF), which is an adaptation of one of the oldest templates from the early works of Sir Henry Head [19]. The image has 4 views of the human body in 2D: coronal (front and back) and sagittal (left and right). The image was modified to demarcate 4 nonoverlapping regions: the front, side head (right), side head (left), and back. For making regions *clickable*, the body image was vectorized and converted into SVG using the Inkscape Editor (version 1.1) [20]. Vectorization produced 4 vectors (beziergons), where each vector has an associated bounding box, which is the tightest fitting rectangle that encloses all points on the vector (Figure 1).

**Figure 2:**
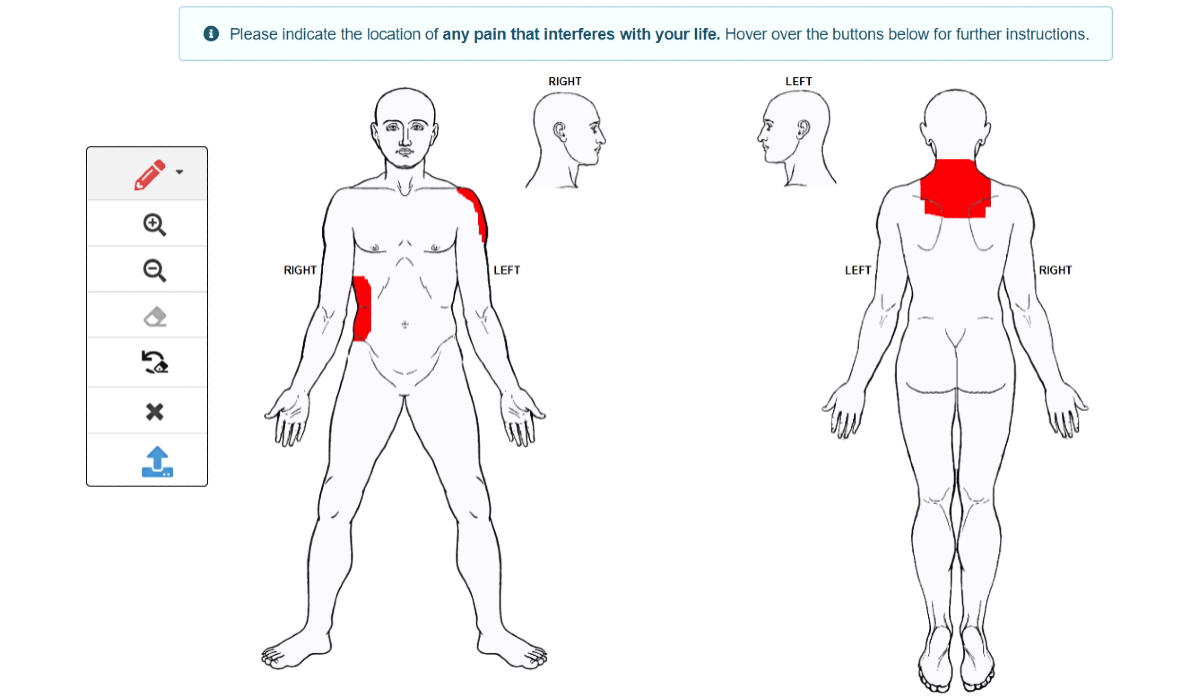
HTML webpage with a 2-column layout. The left column contains all control elements (buttons) and the right column contains the Scalable Vector Graphics body template. The participant can click anywhere on the template (divided into 4 regions) to locate their pain; a pain drawing of body regions, which comprises overlapping squares (rectangles of same width and height) of varying sizes is shown here. These drawings are input to the algorithm.

In order to facilitate data capture from drawings of bodily pain locations, we first developed *Body Pain Map*, a tool using Linux-Apache-Perl-MongoDB–based infrastructure. The HTML5 webpage of the tool consists of a 2-column layout where the right column embeds the SVG body template and the left column contains 7 JavaScript-powered control elements (Figure 2), which are (1) size of rectangular pencil tip (*<select>* element with 3 options, small, medium, and large, signifying red squares of dimensions: 10, 30, and 60). The dimension of the smallest square was based on the smallest width found on the body template (eg, little finger), (2) zoom in (*<button>* element) to allow a closer view, (3) zoom out (*<button>* element) to allow a wider view, (4) Erase (*<button>* element) to remove a previous pain recording, (5) undo eraser (*<button>* element) to restate a previously removed recording, (6) clear (*<button>* element) to remove all recordings, and (7) submit (*<button>* element) to submit drawings for storage in the MongoDB database for analysis and reconstruction purposes. A blank body template can be submitted (eg, when the participant has no bodily pain to draw).

**Figure 3:**
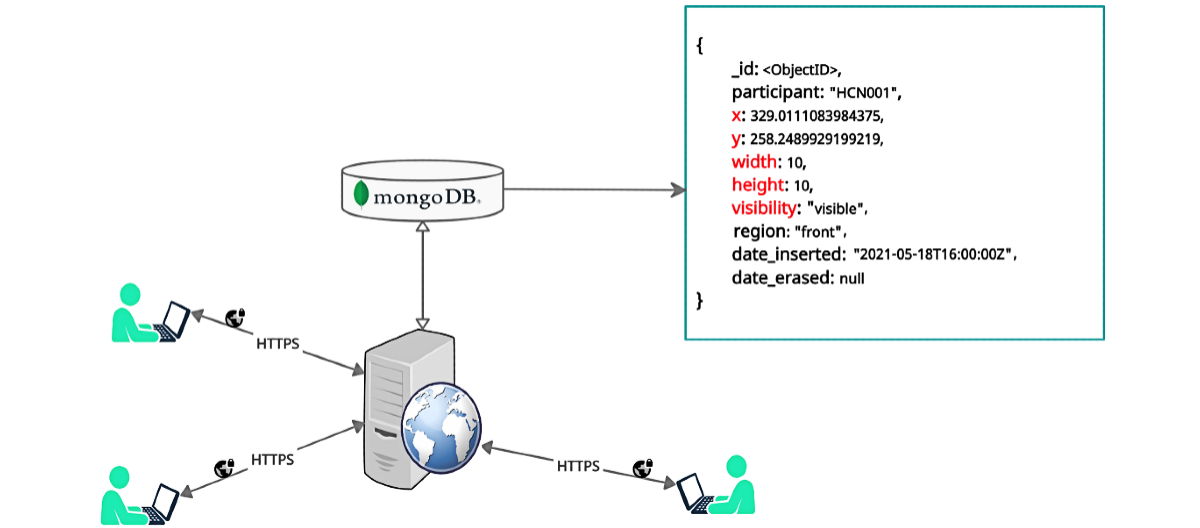
Each participant accesses the Body Pain Map tool through their web browser securely. The pain drawings are captured and stored in a MongoDB database. A sample JSON document of the pain recording (denoting Scalable Vector Graphics <rect> element) is shown as an example with core attributes in the color red.

For making the Body Pain Map easily accessible as a web-based tool, we placed the HTML document on a secure Linux machine running the Secure Socket Layer (SSL)–enabled Apache HTTP web server (Figure 3). The tool can be accessed on the web [21] and tested on modern web browsers (eg, Google Chrome, Mozilla Firefox, and Microsoft Edge).

### Developing the Algorithm to Generate a Pain Frequency Map

#### Overview

The pain frequency map is generated by superimposing several drawings made by the participants using a rectangular pencil tip on a given body template. The drawing from each participant indicates where the pain is located on the body and can comprise multiple overlapping rectangles. The degree to which the rectangles overlap when all drawings are superimposed is of interest. The algorithm described in the *Overlap Computation Algorithm* section generates a pain frequency map that denotes the proportion of overlap in the rectangles between participant drawings. By default, the areas where the proportion of overlap is higher (ie, where pain is more commonly located) are redder in color. Areas with less overlap appear less red. Areas that are colored white are regions within the body template where no participant has drawn.

#### Overlap Computation Algorithm

The key steps in the overlap computation algorithm are (1) data decomposition, (2) creating partitions, (3) merging partitions to create nonoverlapping rectangles, and (4) optimizing nonoverlapping rectangles.

#### Data Decomposition

The first step toward 2D overlap computation is the decomposition of the source data from all participants’ drawings on a given 2D body template.

Let *T* be the total number of participants, *P* (|P| ≤ T) be the set of strings denoting all participants with a drawing, *R* be the set of visible and unique rectangles from all participants in the *front* region of the body template, and *I* be the index set of set R, we assume that all participants in P contribute a drawing each, and as each participant drawing contains at least one rectangle, we define the relationship between rectangles (indexes) and participants as a surjective function *rectToParticipant*:

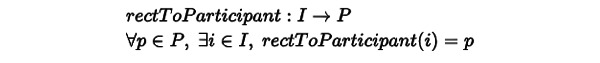



Assuming that each rectangle has attributes *x* (x coordinate of the top left corner), *y* (y coordinate of the top left corner), *w* (width) and *h* (height) we decompose rectangles along the x- and y-axis using the functions *coordX* and *coordY*:

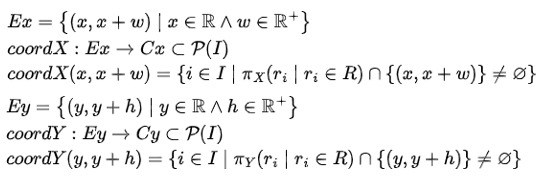



#### Creating Partitions

In this step, we create nonoverlapping partitions along the x- and y-axis. Each partition is an interval window of varying size and represents an area enclosed by at least one rectangle.

Let *E* be the domain and *C* (family of sets) be the range of the function *coordX* or *coordY* depending on the axis.

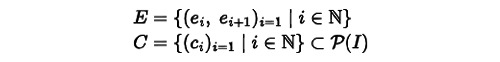



The function *partition* for creating partitions along the x- and y-axis is defined as follows:

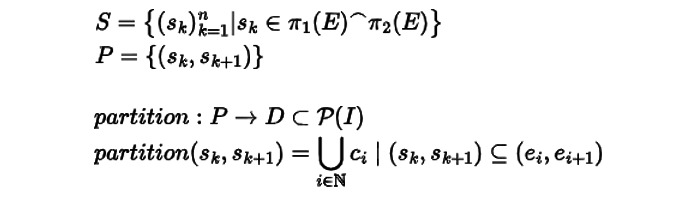



#### Merging Partitions to Create Nonoverlapping Rectangles

This step involves merging the X and Y partitions (nonoverlapping interval windows) to create nonoverlapping rectangles.

Let *Px* and *Dx* (family of sets) be the domain and range of *partitionX* and *Py* and *Dy* (family of sets) be the domain and range of *partition.*

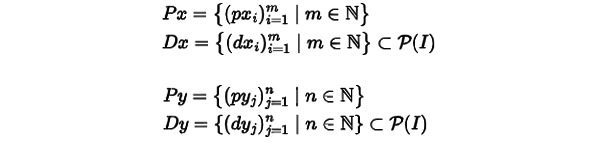



We define *O* (a family of sets of participants *P*) as a set of all overlaps. The function *rectToParticipant* defined earlier is used to map the rectangles to their corresponding participants.

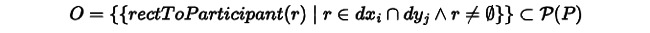



For any *o* (*o* ∈ *O*) the set of nonoverlapping rectangles *R_ME_* can then be defined as

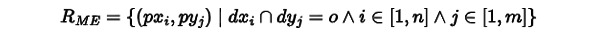



Each element in *R_ME_* represents a unique, nonoverlapping location in the *front* region of the body template and carries an overlap frequency |o| or proportion given by 
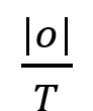
.

#### Optimizing Nonoverlapping Rectangles

In the previous step we generated nonoverlapping rectangles some of which might be adjoined.

This step describes the merging of adjoining rectangles for all the observed overlaps (*O*). The merging of rectangles is important for two main reasons: (1) to optimize the dimensions of the rectangles (ie, as wide or long as possible) and (2) to reduce the size of the output file (SVG) for efficient rendering by the web browser.

Let *r_1_* and *r_2_* be 2 nonoverlapping rectangles associated to the overlap *o* (*o* ∈ *O*).

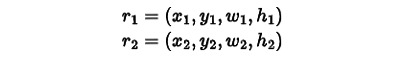



The 2 rectangles *r_1_* and *r_2_* are eligible for horizontal or vertical merging, provided that either of the following conditions is met.

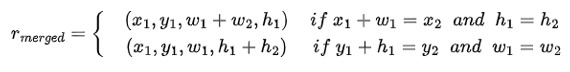



**Figure 4:**
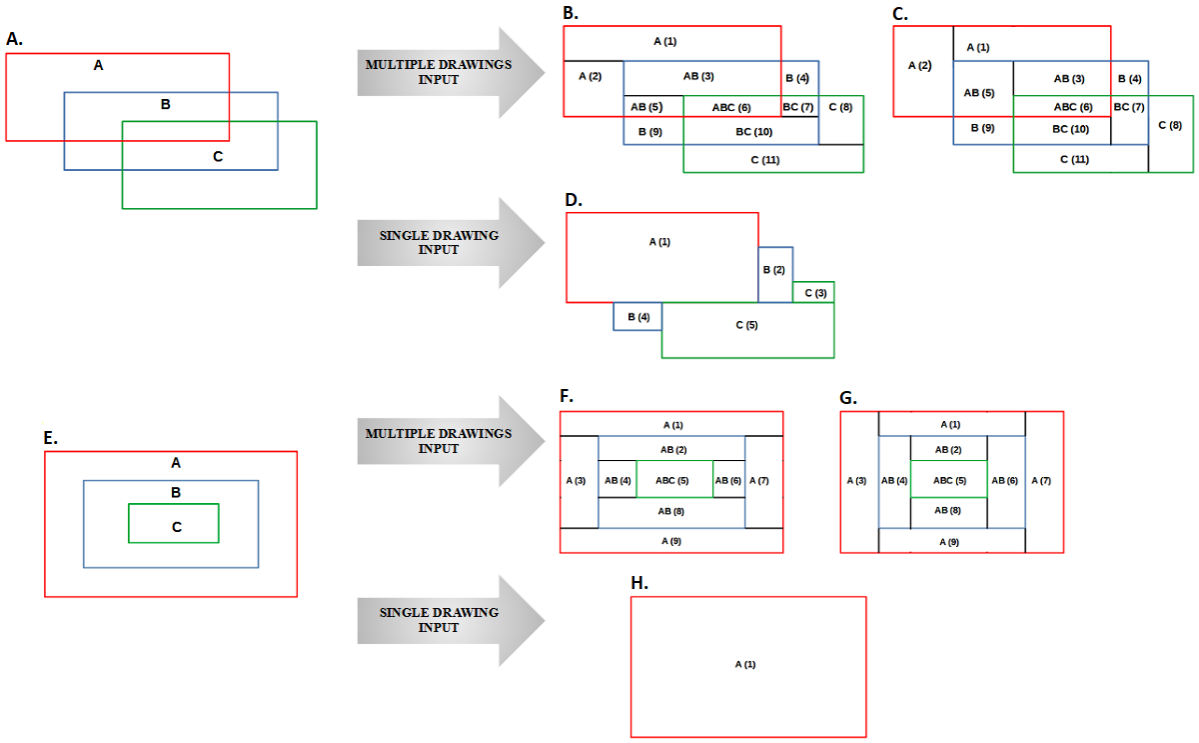
Illustration of the algorithm’s input and output. (A) A, B, and C are individuals who have drawn red, blue and green rectangles that overlap on a body template. (B) These overlapping rectangles are inputted to the algorithm. The output is 11 nonoverlapping rectangles (horizontal layout), named 1 to 11 in parentheses. Each rectangle represents a location with the proportion of pain. For example, A(1) = 1/3 , AB (3) = 2/3, ABC (6) = 3/3. (C) This shows the output from the algorithm in the vertical layout. The choice of layout (horizontal or wider rectangles or vertical or longer rectangles) can be made as per the locations of pain. For the legs, the rectangles might be better visualized when the layout is vertical, whereas horizontal layout is preferred for the abdominal area. (D) The illustration of the algorithm’s output when the input is a single drawing made by one individual (A, B and C are the same individual). In this case, the output is simply 5 nonoverlapping rectangles instead of 11 each with a proportion of 1.0. (E) A, B and C are individuals who have drawn red, blue and green rectangles that overlap in a nested fashion, on a body template. These nested rectangles when inputted to the algorithm produce 9 nonoverlapping rectangles, with (F) showing the horizontal and (G) showing the vertical layout. (H) The demonstration of the output (ie, 1 nonoverlapping rectangle) when the input is a single drawing consisting of nested rectangles. This tends to occur when the individual elects to switch between pencil (tip) size: small, medium and large.

Horizontal merging produced wider rectangles, whereas vertical merging produced longer rectangles (Figure 4).

### Algorithm Analysis Pipeline Construction

For implementing the overlap computation algorithm, we developed a command line workflow in Python programming language release 3.9 [22], which involves 3 steps (scripts) described further. The scripts can be run in either sequential or pipeline mode (using the named pipe command “|”). Each script performs a specific operation and accepts parameters in the form of command line options and arguments. The scripts use some core Python modules that are responsible for reading command line arguments, parsing and validating SVG to be processed, and producing the output for the subsequent step in the workflow. The following scripts can be obtained by contacting the corresponding author (AD):

1. *extract_data.py*: this script is only used if the pain drawings are originally saved as SVG files (a file per participant). The script extracts all the pain recordings denoted by *<rect>* elements with the same width and height attributes. The script produces a CSV output. The columns in the output are (1) *participant*: the identifier of the drawing, (2) *x*: the x coordinate of the top left corner of the rectangle, (3) *y*: the y coordinate of the top left corner of the rectangle, (3) *width*: the width of the rectangle, (4) *height*: the height of the rectangle (same as width), (5) *region*: the region of the body template where the recording belongs (eg, front or back), and (6) *visibility*: the visibility status of the recording (hidden means erased). The script also provides the option of including or excluding *empty* files. These files represent instances when the participant had no pain to draw or indicate on the body template. The script also allows users to extract data from specific body regions (eg, front or back).
2. *compute_overlap.py*: this script implements the overlap computation algorithm to produce nonoverlapping rectangles. Each rectangle represents a unique location on the body template. The script accepts input in CSV format with columns, namely, *participant*, *x*, *y*, *width*, *height*, and *region*. The script returns a CSV output where rows are nonoverlapping rectangles, and the columns (rectangles’ attributes) are (1) *x*: the x coordinate of the top left corner of the rectangle, (2) *y*: the y coordinate of the top left corner of the rectangle, (3) *width*: the width of the rectangle, (4) *height*: the height of the rectangle, (5) *area*: the area of the rectangle, (6) *overlap*: the identifiers of drawings that overlap, (7) *overlap_frequency*: the number of identifiers that overlap, and (8) *overlap_proportion*: the proportion of identifiers that overlap. The proportion is calculated from the total number of drawings, which may include empty drawings. The script also allows users to filter the output by providing the range for overlap (frequency) and thresholds for the width and height of the rectangles.
3. *plot_heatmap.py*: this script plots the nonoverlapping rectangles on the blank SVG body template and generates a pain frequency map (heat map) as an HTML file. The color of the pain frequency map can be specified either by its native name or as a hexadecimal color code (default #ff0000 or red). The intensity (shade or gradient) of the participant overlap on the pain frequency map is displayed using the opacity attribute of an SVG element. Opacity is any number strictly between 0 and 1. For optimum color coding, the opacity for each output rectangle is calculated as follows:

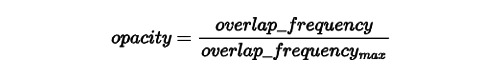

### Data Generation for Algorithm Implementation

#### Simulation

We examined the performance of the algorithm’s Python script *compute_overlap.py* in 2 separate simulations on a machine (Intel Xeon[R] Silver 4110 CPU@2.10GHz and 16-GB RAM) running Ubuntu 18.04. Given an XY plane of dimensions 1000×1000 with the origin at (0,0), let *X* and *Y* be the set of all natural numbers on the x- and y-axis. The set of all ordered pairs *P* is denoted by the Cartesian product *X×Y* of sets *X*, *Y*.

#### Simulation 1

We assumed that the drawings from every individual consisted of a single rectangle (analogous to a single mouse *click* on the body template). This situation is extremely unlikely; however, the purpose of this simulation was to test the ability of the algorithm to compute an overlap, given a set of overlapping rectangles.

A total of 10 data sets were generated for this simulation. The first data set consisted of 10,000 rectangles, and for each consecutive data set, the number of rectangles was increased by 10,000. For each data set, the coordinates of the origin of the rectangles were sampled without replacement from the set of ordered pairs (*P*), and the dimensions (width and height) were sampled with replacement from a sequence starting at 10 and ending at 100 (incremental step is 10).

#### Simulation 2

We assumed that a typical participant drawing of pain locations consisted of 100 rectangles (equivalent to 100 mouse *clicks* on the body template). For this simulation, we sought to assess performance with an increasing number of participants.

A total of 10 data sets were generated for this simulation. The first data set consisted of 100 participants, and for each consecutive data set, we increased the number of participants by 100. For each participant, the origin of the rectangles was sampled without replacement from the set of ordered pairs (*P*), and the dimensions (width and height) were sampled with replacement from a sequence starting at 10 and ending at 100 (incremental step is 10).

Each simulation produced 10 CSV files for analysis by the algorithm. The CSV file consisted of the columns, namely, *participant*, *x*, *y*, *width*, *height*, and *region*. For both simulations, there was only one region (the XY plane); therefore, the *region* was simply labeled *xy-plane*.

#### Actual

Finally, we used the digital pain drawings from 2 clinical data sets; the first data set (data set 1) consisted of 23 individuals who were screened for a clinical drug trial (ISRCTN68734605) [23], and the second data set (data set 2) comprised 30 participants with chronic back pain who were recruited into a separate study [24]. The pain drawings were stored as MongoDB documents (Figure 3), which were organized into subdocuments ordered by the date and time of insertion. The virtual pencil tip used for the drawings was a square, an SVG *<rect>* element, where the width and height attributes were the same (Figure 2).

## Results

### Simulation

**Figure 5:**
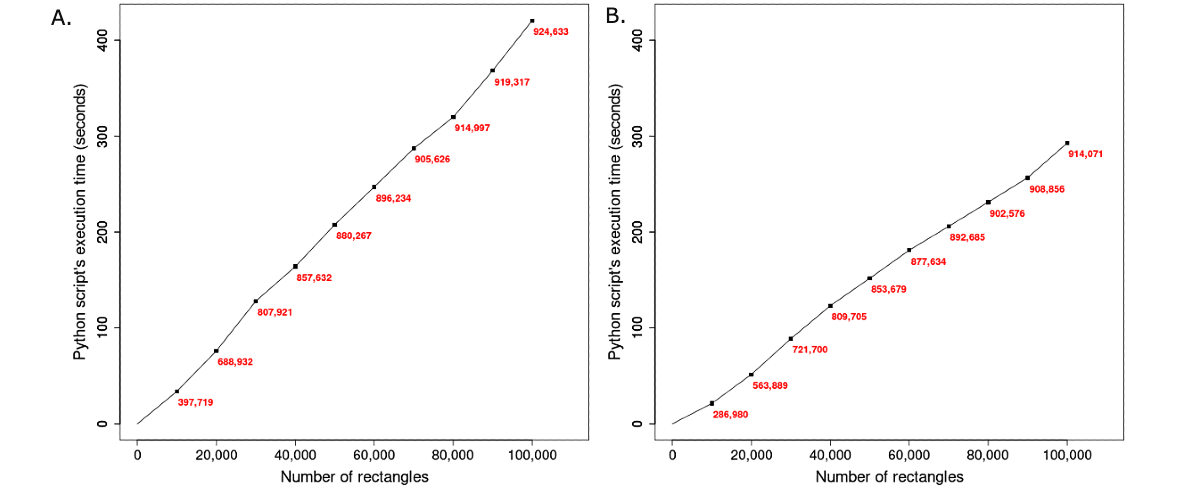
Simulations to assess the performance of the algorithm’s Python script – compute_overlap.py. The y-axis shows the execution time of the script in seconds and the x-axis shows the number of rectangles handled by the script. The output from the script, the number of nonoverlapping rectangles is shown in red. The plot (A) shows the script’s performance on the data sets from simulation 1 where the number of rectangles per participant was set to 1, and plot (B) shows the performance on the data sets generated in simulation 2 where the number of rectangles per participant drawing was set to 100.

The simulations were carried out to assess the performance of the algorithm’s Python script *compute_overlap.py*. The simulations reveal that the script’s execution time (seconds) increases linearly as a function of the number of input rectangles (Figure 5), up to 400 seconds for 100,000 rectangles (simulation 1). Assuming that a typical participant drawing consists of 100 rectangles, the execution time for 1000 participant drawings is 300 seconds (simulation 2).

### Actual

We first extracted all drawings (<rect> elements with the same width and height attributes) from the 2 data sets stored in the MongoDB database into CSV files. Data set 1 produced 4016 and data set 2 produced 4167 recordings, respectively, sorted by the participant identifier and the date and time of insertion. The characteristics of the participants from the 2 data sets pertaining to the *Body Pain Map* exercise are summarized in Table 1.

For generating pain frequency maps, we only used visible and unique (nonidentical) recordings from the 2 clinical data sets. Identical recordings from the same participant (*<rect>* elements with complete overlay) were filtered based on the recording’s last visibility status. If visibility was hidden (meaning erased), the recording was excluded. This exercise produced 2 CSV files (one file per data set) with 3242 and 3993 rows, respectively, signifying unique and visible recordings across all 4 regions, namely, front, side (right), side (left), and back as summarized in Table 2.

The CSV data sets were independently processed by the Python script *compute_overlap.py* on a machine (Intel(R) Xeon (R) Silver 4110 CPU @ 2.10GHz and 16 GB RAM) running Ubuntu 18.04*.* The script processed the first CSV data set in 4 seconds and produced 6653 nonoverlapping rectangles with an overlap frequency between 1 and 8. The second CSV data set was processed in 7 seconds and produced 6010 nonoverlapping rectangles with an overlap frequency ranging from 1 to 21. Each nonoverlapping rectangle represents a unique location on the 2D body template and carries an overlap frequency, which denotes the number of participants with pain at that location.

**Figure 6:**
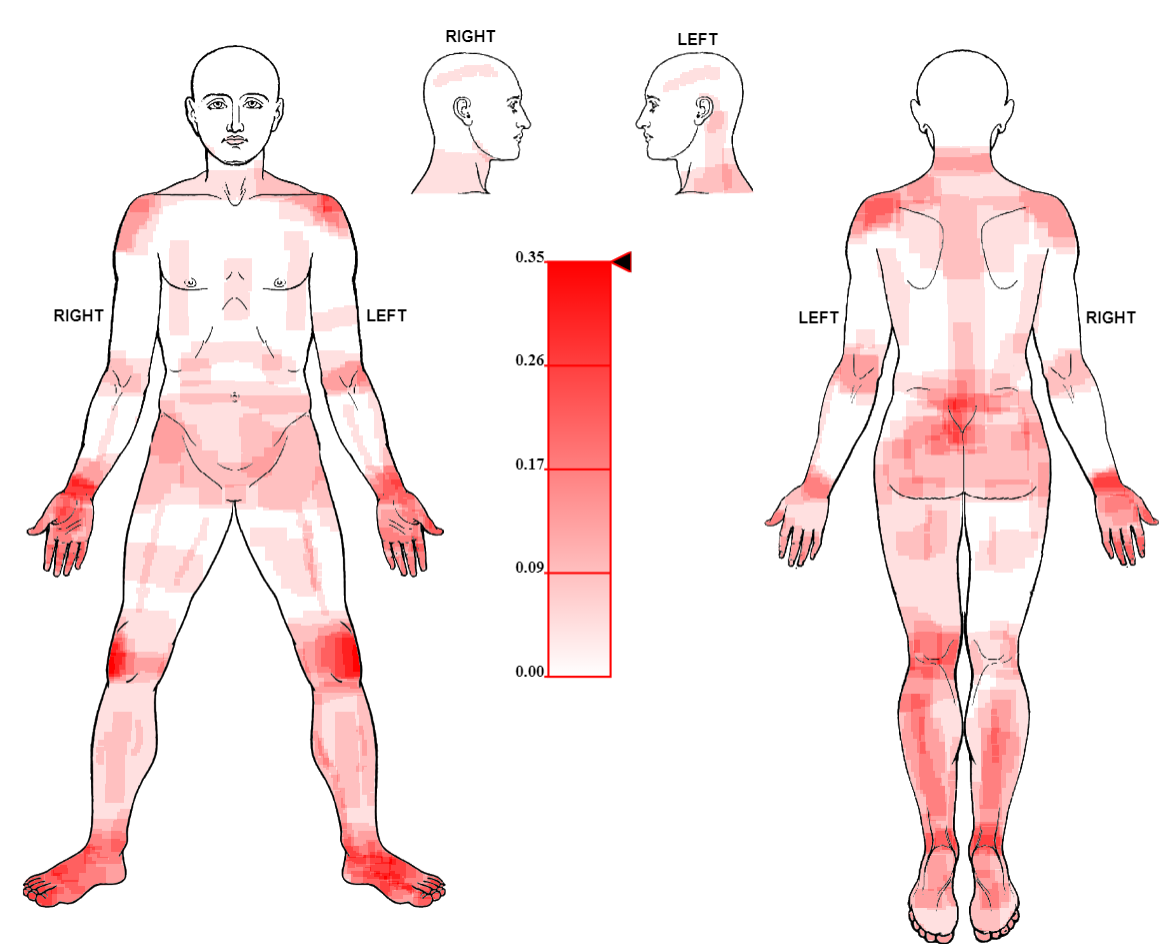
Illustration of the pain frequency map produced from freehand pain drawings (data set 1) obtained from patients (N=23) who were screened for a clinical drug trial. In the interactive map (HTML format), the user can slide the black pointer on the gradient bar (shown in the middle) to view locations based on the overlap threshold (ie, <= overlap proportion) and save the map as a PNG file.

**Figure 7:**
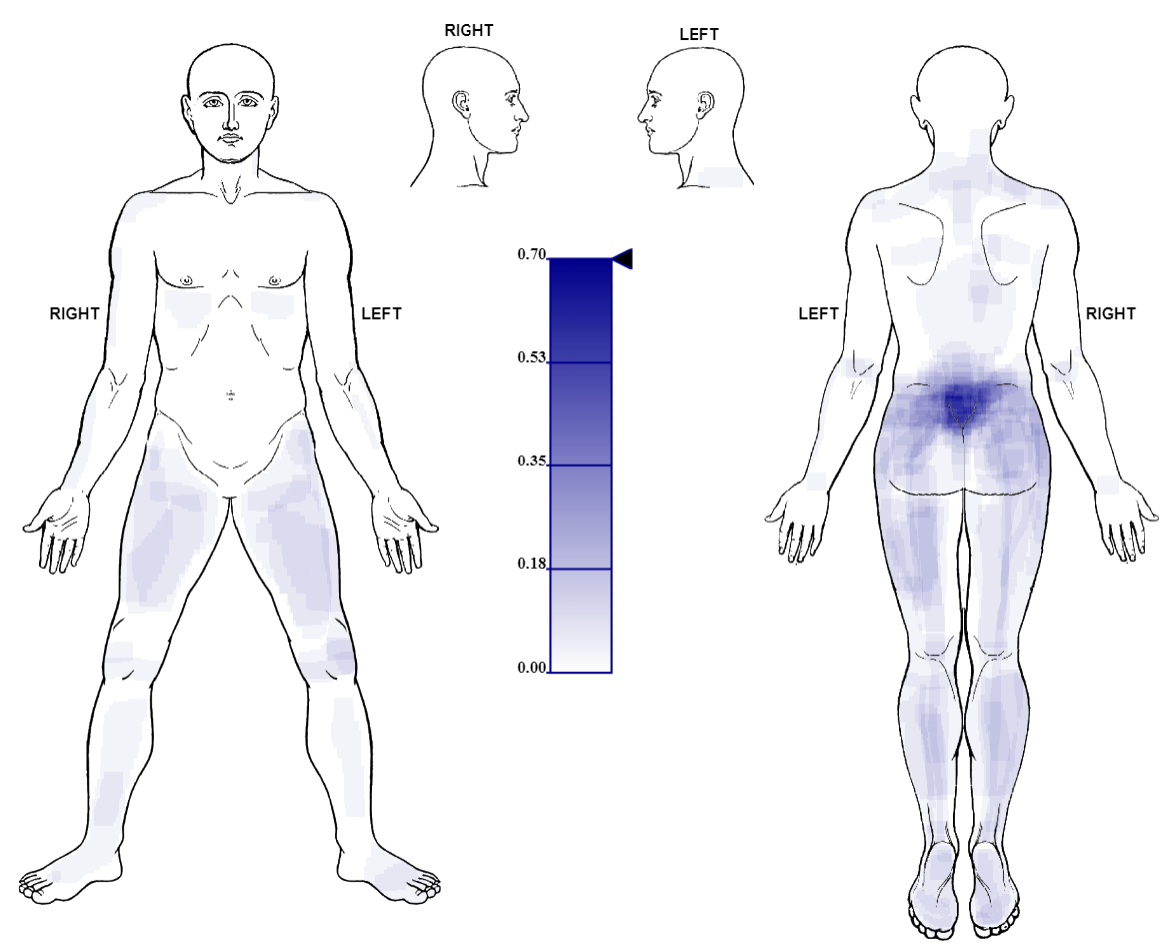
Illustration of the pain frequency map produced from freehand pain drawings (data set 2) obtained from patients (N=30) diagnosed with chronic primary back pain.

The nonoverlapping rectangles for the 2 clinical data sets were subsequently plotted on the body template using the Python script *plot_heatmap.py*, and a pain frequency map was generated for data set 1 (Figure 6) and data set 2 (Figure 7).

**Table 1:** Pencil and eraser clicks made by the participants from the 2 clinical data sets while using the Body Pain Map tool.

Characteristic			Data set 1		Data set 2
Participants, N			23		30
**Clicks, mean (SD)**					
	Pencil^a^	174.6 (208.5)		138.9 (108.2)	
	Eraser^b^	16.7 (32.7)		1.5 (4.2)	
Visible and unique pencil clicks, mean (SD)			141.0 (176.5)		133.1 (105.5)
Mean pencil size^c^, mean (SD)			26.6 (10.3)		25.2 (6.0)

^a^Each pencil click denotes a recording of the pain location and creates a Scalable Vector Graphics *<rect>* element with the same width and height attributes (a square).

^b^The eraser click hides the previously inserted Scalable Vector Graphics *<rect>* element.

^c^On the basis of the visible and unique pencil clicks to record (draw) pain locations.

**Table 2:** Visible and unique pencil recordings made in each region for 2 clinical data sets^a^.

Characteristic		Data set 1	Data set 2
Recordings, N		3242	3993
**Region, n (%)**			
	Front	1381 (42.6)	800 (20.0)
	Side (right)	80 (2.5)	0 (0.0)
	Side (left)	103 (3.2)	11 (0.3)
	Back	1678 (51.7)	3182 (79.7)

^a^Data are derived from the Scalable Vector Graphics drawings and provided to the algorithm’s Python scripts to generate a pain frequency map.

## Discussion

### Principal Findings

Drawing pain locations on body templates is widely and increasingly being used in research and pain clinics. These are frequently part of questionnaires (eg, the Brief Pain Inventory [25]). The topography of bodily pain is often summarized as a pain frequency map but relies on digitization of paper drawings, which is labor intensive and may be infeasible for larger studies [26]. Although freehand digital drawing tools are available to capture pain locations, they are often restricted to specific body templates [6]. Other tools parcellate the body (eg, the CHOIR Body Map [7] and Michigan Body Map [13]), which allows easy generation of pain frequency maps but lacks the resolution required.

In this paper, we first described the Body Pain Map [21], a web-based platform used to capture freehand pain drawings as SVG *<rect>* elements and then thoroughly described the algorithm to generate a pain frequency map from the drawings. We further described the implementation of the algorithm as a Python-based analysis pipeline and tested its performance in 2 different simulations. We demonstrated the utility of the algorithm by producing pain frequency maps (Figure 6 and Figure 7) from freehand drawings obtained from 23 patients screened for a clinical drug trial (ISRCTN68734605) [23] and 30 patients diagnosed with chronic primary back pain [24].

The key advantage of this algorithm is that it can handle data from any undemarcated body template. In other words, the body template does not need to be divided using a mesh with a fixed number of cells (eg, GeoPain, a body surface map rendered on a 3D manikin [9] and Manchester Digital Pain Manikin [15]) or demarcated into regions with anatomical labels (eg, CHOIR [7]). Only the outline or perimeter of the body template requires demarcation. The label-free and grid-free approach is unbiased and allows the participants to freely draw the locations of their bodily pain, closely mimicking the experience of drawing on paper. Although the user is unlikely to draw in the entire space, given the nature of freehand drawing, they can draw repeatedly in the same area. This results in redundant data, which are not usually of interest to most clinician researchers. Our algorithm processes raw (source pixel) data in grid-free space to address this difficulty, and in doing so, it also achieves lossless compression.

The algorithm accepts a single CSV (plain text) file with pain recordings captured as rectangles and produces nonoverlapping rectangles. Each nonoverlapping rectangle is a unique location on the body template and carries an overlap frequency, which denotes the number of participants with pain in that location. The output, when transformed into an HTML file, can be feasibly rendered on modern web browsers (eg, Google Chrome, Mozilla Firefox, or Microsoft Edge) or printed at the desired resolution for publishing as we have shown for 2 clinical data sets. The algorithm also contains features to optimize the display of the pain frequency map. Rectangles can be produced either in a horizontal (wider) or vertical layout (longer; Figure 4). For example, for the leg, the rectangles may be better visualized with a vertical layout, whereas a horizontal layout is preferred for wider regions such as the abdomen.

As the individual can draw freely on the body template, the output (ie, nonoverlapping rectangles) produced by the algorithm is highly granular. Given the required spatial information (x-y coordinates within the body region), it is possible to retrospectively create a grid and reassign the cells and anatomical labels as required. The output from the algorithm can be subjected to coordinate transformation using linear algebra and labeled to allow harmonization with other body atlases or templates with anatomical labels (eg, arms and legs) in nature [7]. The unbiased output can also be trained to provide demarcations for a given pain disorder within any 2D body template. The demarcations or boundaries of areas for an anatomical label (eg, shoulder) may also be determined empirically through feedback from any group of individuals. It is also possible to acquire drawings from gender-specific body templates provided by the Hannover Medical School [8] and subsequently use the coordinate transformation to harmonize the algorithm’s output on a gender-neutral template for comparisons between sexes.

### Limitations

The algorithm generates nonoverlapping rectangles without any knowledge of the region boundaries within the 2D body template. Hence, some rectangles in the output may fall partly outside these regions. This overflow occurs when drawings include the border (or are close to the border) of the body regions. For generating pain frequency maps, this is not a problem because rectangles falling outside the regions can be easily masked (hidden) using SVG. For other analysis purposes, the problem can be feasibly mitigated by approaches such as (1) dividing the body template into small regions and applying bounding box correction to filter output nonoverlapping rectangles by region or (2) creating a *null* drawing that delicately fills the entire body template, ensuring that the *<rect>* elements are within the regional boundaries. With this approach, the *null* drawing is added to actual participant drawings, and the algorithm is executed on the combined data set. In the resulting output, only the nonoverlapping rectangles that are common to both the *null* drawing and the actual drawings are retained, and the rest (overlap frequency is 1) are discarded.

The algorithm is optimized to generate pain frequency data only for the locations of the body template on which at least one participant has drawn. Adding the *null* drawing would allow representation (in the pain frequency map) of body locations within the template that no participant has drawn and may also be required for inferential statistics. As the *null* drawing is specific to a body template, it does not have to be recreated, and the approach can be applied to prospective data sets (drawings), provided they are acquired using the same body template.

### Comparison With Prior Work

Software tools (R package, CHOIR Body Map [27]) have recently been developed to generate co-occurrence maps. The map shows the number of times 2 locations on the body template are endorsed together by patients in a data set. However, these tools are only applicable to the CHOIR body template [7], where the participant can only click on 74 predefined locations.

Generating a pain frequency map for body templates with either demarcations or grid is straightforward because the locations are predefined and fixed, and it is a case of deducing the number of participants that selected each location [14].

Studies in which a nondemarcated and grid-free body template was used for freehand drawings used customized tools for the simultaneous analysis of their drawings. These tools are not freely available in the public domain [16-18]. Other studies [28,29] converted their drawings (originally saved as PNG images) into NIfTI format [30] and analyzed them using image-processing tools provided by the Functional Magnetic Resonance Imaging of the Brain (FMRIB) Software Library [31].

The primary purpose of our algorithm and other methods used in previous studies is to generate pain frequency maps, which requires simultaneous analysis of all freehand drawings provided by participants. Previous studies have stored freehand pain drawings as bitmap images (eg, PNG); therefore, the generation of a pain frequency map requires the extraction and analysis of all pixels [16-18,28,29].

Our algorithm processes drawings in which pain locations are indicated using rectangles (the pencil tip is a square, which is fundamentally a rectangle). This has several advantages, such as the fact that the input from the drawings can be simply stored as a CSV (plain text) file because all locations are represented as rectangles with attributes *x*, *y*, *width,* and *height*. This is also more efficient than the storage and extraction of pixels from the drawings. The output also comprises rectangles and can be stored as CSV files for statistical analyses and visualized using images (eg, SVG and PNG). We used SVG because it allows the reconstruction and visualization of the input and output rectangles at the desired resolution. CSV storage also facilitates the merging of several independent data sets acquired using the same body template for a combined analysis.

### Conclusions

Body maps have long been used in research and clinical practice to facilitate communication between pain and other sensations [32]. The choice of a drawing tool or body part selection depends on the nature of the research question and the participants. It is important to clinically validate any digital tool used to capture the topography of body sensations [33]. Our algorithm is primarily developed to render pain frequency maps for efficient display and printing, but the output (ie, nonoverlapping rectangles) can be readily subjected to statistical analyses (eg, statistical comparisons of pain frequency maps between different patient cohorts or the same patient cohort over multiple time points). Although we chose to digitize and use a specific body template in our study, the algorithm described can process any number of freehand drawings on any 2D body template to produce a pain frequency map. The nonoverlapping rectangles generated by the algorithm can be labeled anatomically or mapped onto a grid to facilitate analyses and harmonization with other body templates.

Our freehand pain drawing tool (Body Pain Map [21]) uses resolution-independent and XML document object model–based SVG technology. However, our algorithm can also generate pain frequency maps from drawings created using other technologies (eg, HTML5 *<canvas>* element), provided that the pain locations are captured as rectangles and the location attributes (ie, *x*, *y*, *width,* and *height*) are accessible.

As the algorithm has already been implemented as a Python command line workflow, it is possible to schedule an automated pain frequency map construction through the cron daemon (Linux environment) and filter and visualize the output using several criteria (eg, region, overlap frequency, width, and height of rectangles). The Python scripts can be obtained by contacting the corresponding author (AD).

We envisage the use of the algorithm in clinical or research settings to facilitate fine-grain comparisons of human pain anatomy between disease diagnosis or disorders or as an outcome metric to guide the monitoring or discovery of treatments.

## Abbreviations

CHOIR: Collaborative Health Outcomes Information Registry
FMRIB: Functional Magnetic Resonance Imaging of the Brain
SSL: Secure Socket Layer
SVG: Scalable Vector Graphics
